# Adult clear cell sarcoma of the kidney: A case report

**DOI:** 10.1186/1471-2490-6-11

**Published:** 2006-04-03

**Authors:** Ali R Kural, Bulent Onal, Hamdi Ozkara, Cansel Cakarir, Inci Ayan, Fulya Y Agaoglu

**Affiliations:** 1 Department of Urology, University of Istanbul, Cerrahpasa School of Medicine, Istanbul, Turkey; 2 Department of Pathology, University of Istanbul, Cerrahpasa School of Medicine, Istanbul, Turkey; 3 Division of Pediatric Oncology, University of Istanbul, Institute of Oncology, Istanbul, Turkey; 4 Department of Radiation Oncology, University of Istanbul, Istanbul School of Medicine, Istanbul, Turkey

## Abstract

**Background:**

Clear cell sarcoma of the kidney (CCSK) in adults is extremely rare. Optimal treatment of adult patients with CCSK remains unclear.

**Case presentation:**

A 22-year-old man presented with a 2-month history of left flank pain. A color duplex sonography revealed a hypervascular, heterogeneous renal mass. Abdominal and pelvic computerized tomography showed a heterogeneous mass originating from the lower pole of the left kidney and infiltrating to the psoas muscle. Further evaluation including bone scan did not demonstrate any evidence of metastases. A left radical nephrectomy with hilar lymphadenectomy through an intraperitoneal approach with an anterior subcostal incision was performed. The histopathological diagnosis of the mass was a clear cell sarcoma of the kidney. No lymph node metastases were found. Concomitant chemo-radiotherapy was performed. Therapy-related serious side effects were not observed. There was no evidence of local recurrence or metastases during the following twenty-four months after therapy.

**Conclusion:**

We believe that the combination therapy is efficacious for preventing the local recurrence and distant metastases. Accurate diagnosis is very important and therapy must also include doxorubicin regardless of the disease stage in adult patients with CCSK.

## Background

Clear cell sarcoma of the kidney (CCSK) is an uncommon pediatric neoplasm distinct from Wilms tumor and also usually tends to metastasize to bones being different from Wilms tumor [1].

CCSK in adults is extremely rare. Optimal treatment of adult patients with CCSK remains unclear. Surgery, radiotherapy and chemotherapy are combined or used separately [2-4]. Advanced clear cell renal carcinoma is highly resistant to traditional cytotoxic chemotherapeutic drugs, and chemotherapy alone has yielded disappointing responses in patients with renal cell carcinoma [5]. However, Argani and colleagues declared that doxorubicin, regardless of disease stage, was an effective drug in pediatric patients with CCSK [1]. Thus, its differentiation from clear cell renal carcinoma and undifferentiated adult renal neoplasm including sarcomatoid renal cell carcinoma is especially important in adult patients [6].

Herein, we report a 22-year-old patient with clear cell sarcoma of the left kidney, who was managed with radical nephrectomy, radiotherapy and chemotherapy. We think that our case report might provide additional information about the treatment of CCSK in adults.

## Case presentation

**Figure 1:**
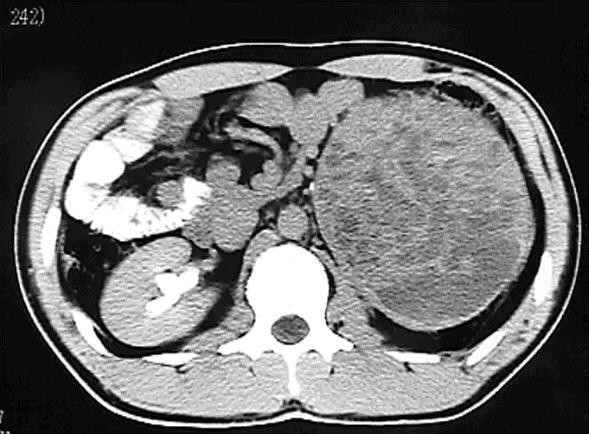
Computerized tomography shows a heterogeneous mass originating from the lower pole of the left kidney and infiltrating to the psoas muscle.

A 22-year-old man presented with a 2-month history of left flank pain. A color duplex sonography revealed a hypervascular, heterogeneous renal mass 125 × 156 × 195 mm. in diameter. Abdominal and pelvic computerized tomography showed a heterogeneous mass originating from the lower pole of the left kidney and infiltrating to the psoas muscle (fig. 1). Further evaluation including bone scan did not demonstrate any evidence of metastases. A left radical nephrectomy with hilar lymphadenectomy through an intraperitoneal approach with an anterior subcostal incision was performed. The patient was discharged from hospital on postoperative day 5 without any complication.

**Figure 2:**
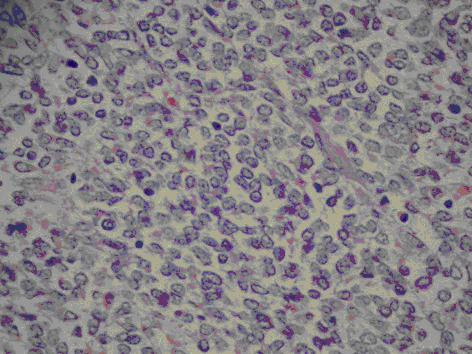
Classic pattern of clear cell sarcoma of the kidney characterized by cells with fine nuclear chromatin, pale cytoplasm and indistinct cell borders forming nests separated by a fibrovascular stroma. (H&E × 400)

Histopathological examination revealed that classic pattern of clear cell sarcoma of the kidney consisted of cells with fine nuclear chromatin, pale cytoplasm and indistinct cell borders forming nests separated by a fibrovascular stroma (fig. 2). Stain for cytokeratin was negative while tumor cells were positive for vimentin. The histopathological diagnosis of the mass was a clear cell sarcoma of the kidney. No lymph node metastases were found. Final pathologic stage was T3aN0M0 and accepted as stage 2 according to updated National Wilms Tumor Study 5 definition. Concomitant chemo-radiotherapy was performed. The patient underwent adjuvant radiotherapy to the left nephrectomy bed (4950 cGy). As adjuvant chemotherapy, Actinomycin (1.2 mg/m2) with Vincristine (2 mg/m2) and Doxorubicin (30 mg/m2) with Vincristin (2 mg/m2) were administered subsequently for one day every 3 weeks during one-year period. Therapy-related serious side effects were not observed. There was no evidence of local recurrence or metastases during the following Twenty-four months after therapy.

CCSK is frequently confused with Wilms tumor in children. However, Argani and colleagues reported the striking differences between clear cell sarcoma and Wilms tumor [1]. The large size, areas of necrosis, a mucoid structure, and the cyst formation are the important gross features of this neoplasm. Stain for cytokeratin is negative while tumor cells are positive for vimentin in clear cell sarcoma of the kidney. It usually tends to metastasize to bones being different from Wilms tumor. It is declared that in pediatric patients the treatment with doxorubicin, the presence of necrosis, the stage of the disease, and the age of the patient at diagnosis were the significant prognostic factors for survival. These findings had been obtained from the records of the pediatric patients [1].

CCSK is extremely rare neoplasm in adults. Amin and colleagues reviewed four new and eight previous cases of CCSK occurring in adult patients. They reported that clinic and pathologic features of CCSK did not differ significantly between adult and pediatric patients [6]. They also noted that CCSK was negative for most markers except for vimentin and this finding was helpful to distinguish it from undifferentiated adult renal neoplasm including sarcomatoid renal cell carcinoma [6].

Optimal treatment of adult patients with CCSK still remains unclear. Surgery, radiotherapy and chemotherapy are combined or used separately. Benchekroun et al reported a patient who underwent surgery followed by combination chemotherapy with cisplatin and doxorubicin and had no metastases 4 years later, but the metastases occurred within months in two patients who did not receive chemotherapy or radiotherapy after surgery [2]. In addition, Bhayani et al noted that a patient treated with surgery and combination chemotherapy (actinomycin, vincristine and doxorubicin) was disease-free at 12 months postoperatively [3]. In a report recently published by Rosso and colleagues, they treated a patient with surgery alone and stated that CCSK has a high resistance against radiation and chemotherapy [4].

Advanced clear cell renal carcinoma is highly resistant to traditional cytotoxic chemotherapeutic drugs and especially interleukin-2 and interferon alpha, along with selected chemotherapeutic agents have proven to be the most effective, with response rates averaging about 20 percent [5]. However, Argani and colleagues declared that doxorubicin, regardless of disease stage, was an effective drug in CCSK and also pointed that the survival rate was 98% and 69% for the pediatric patients with stage 1 disease and for all stages, respectively [1]. Thus, its differentiation from clear cell renal carcinoma and undifferentiated adult renal neoplasm including sarcomatoid renal cell carcinoma is especially important in adult patients. Our 22-year-old male patient received radiotherapy and combination chemotherapy with actinomycin, vincristine and doxorubicin after radical nephrectomy and lymph node dissection. Twenty-four months following surgery and concomitant chemoradiotherapy there was no evidence of local recurrence or metastases.

## Conclusion

We believe that the combination therapy is efficacious for preventing the local recurrence and distant metastases. As Argani and colleagues suggested in pediatric patients, accurate diagnosis is very important and therapy must also include doxorubicin regardless of the disease stage in adult patients with CCSK.

## Abbreviations

CCSK: Clear cell sarcoma of the kidney

## Competing interests

The author(s) declare that they have no competing interests.

## Authors' contributions

Dr.s ARK and BO have provided the patients and performed the radical nephrectomy, HO has reviewed the charts, CC has evaluated the pathology specimen, IA and FYA have performed the concomitant chemo-radiotherapy. All authors read and approved the final manuscript.

## Pre-publication history

The pre-publication history for this paper can be accessed here:
